# Variation in maternal sensitivity and the development of memory biases in preschoolers

**DOI:** 10.3389/fnbeh.2023.1093619

**Published:** 2023-02-16

**Authors:** Anne Rifkin-Graboi, Stella Tsotsi, Nadhrah Syazwana, Mary C. Stephenson, Lit Wee Sim, Kerry Lee

**Affiliations:** ^1^Office of Educational Research, Centre for Research in Child Development, National Institute of Education, Nanyang Technological University, Singapore, Singapore; ^2^PROMENTA Research Centre, Department of Psychology, University of Oslo, Oslo, Norway; ^3^Centre for Translational MR Research, Yong Loo Lin School of Medicine, National University of Singapore, Singapore, Singapore; ^4^Centre for Educational and Developmental Sciences, Department of Early Childhood Education, Faculty of Education and Human Development, Education University of Hong Kong, Hong Kong, Hong Kong SAR, China

**Keywords:** maternal sensitivity, adversity, memory, hippocampus, gender, development, conditional adaptation, emotion

## Abstract

**Introduction:**

Links between maternal sensitivity, hippocampal development, and memory abilities suggests early life insensitive care may shape structures and schemas influencing future decisions and stress management, biasing children to negative information. While it is possible that this pattern of neurodevelopment may have adaptive consequences, for example, preventing children from encountering untoward experience with future adversity, it may also leave some children at risk for the development of internalizing problems.

**Methods:**

Here, in a Two Wave Study, we examine whether insensitive care predicts sub sequentially assessed memory biases for threatening (but not happy) stimuli in preschoolers (*n* = 49), and if such relations cut across different forms of relational memory, i.e., memory for relations between two “items,” between an “item” and its spatial location, and an “item” and its temporal sequence. In a subset (*n* = 18) we also examine links between caregiving, memory, and hippocampal subregion volume.

**Results:**

Results indicate no main or interactive influence of gender on relational memory. However, insensitive caregiving predicted the difference between Angry and Happy memory during the Item-Space condition (*B* = 2.451, se = 0.969, *p* = 0.014, 95% CI (0.572, 4.340)], as well as memory for Angry (but not Happy) items [*B* = −2.203, se = 0.551, *p* < 0.001, 95% CI (−3.264,−1.094)]. Memory for the difference between Angry and Happy stimuli in the Space condition associated with larger right hippocampal body volumes (Rho = 0.639, *p* = 0.004). No relations were observed with internalizing problems.

**Discussion:**

Results are discussed with reference to developmental stage and in consideration of whether negative biases may serve as an intermediate factor linking early life insensitive care and later socioemotional problems including an increased incidence of internalizing disorders.

## 1. Introduction

Insensitive caregiving behavior occurs when a caregiver does not attend to or respond to a child’s signals in a timely manner and/or in a way that is appropriate to the situation, developmental stage, and expectable needs for security, comfort, exploration, and autonomy. As such intrusiveness, neglect, and rejection may be aspects of insensitive behavior. While all caregivers are likely to periodically exhibit insensitive behavior, at least in mild forms, consistent sensitivity is considered a hallmark of the “sensitive caregiver” (see Ainsworth, 1969). Although behavior that is abusive and fear invoking is clearly not sensitive, many caregivers are likely to behave in ways that are insensitive but decidedly not abusive. Indeed, a large proportion of children are likely to experience insensitive care (see De Wolff and Van Ijzendoorn, 1997; Madigan et al., 2006; Frankenhuis and Amir, 2022), which is itself a risk for the development of internalizing disorders (Cooke et al., 2022). As such, a better understanding of the mechanisms behind this association could allow for new targets for intervention, prior to the development of clinical disorder, with potential benefit for a large proportion of children.

One possible mechanism linking insensitive caregiving to offspring psychological health involves relational memory, or the ability to remember relations between forms of information. Relational memory, and the similar construct of associative memory, are considered integral to episodic (Olsen et al., 2012), and especially autobiographical information, as well as future oriented thinking (Richmond and Pan, 2013). As such, it may not be surprising that relational memory is underpinned by a brain region [i.e., the hippocampus (Hannula and Ranganath, 2008)], frequently implicated in mood disorders and biases toward negative information (Price and Duman, 2020).

Indeed, attentional preferences for negative information and (negative) mood-congruent-memory biases are often thought to help sustain depressed mood (LeMoult and Gotlib, 2019). That is, attention to and the enhanced remembering of negative information may make it difficult for depressed individuals to incorporate and learn from new mood-incongruent information, ultimately biasing experienced-based schemas guiding thought and behavior. Relevant to the current paper, mood-congruent-memory may be most apparent in tasks requiring elaboration (Faul and LaBar, 2022), such as relational memory. This is interesting to consider alongside work indicating that depression also associates with memory deficits (LeMoult and Gotlib, 2019), perhaps suggesting that even simple Relational Memory tasks may be susceptible to bias amongst depressed individuals.

Importantly, insensitive caregiving, and related constructs, are also linked to hippocampal development (Rao et al., 2010; Luby et al., 2012; Rifkin-Graboi et al., 2015; Bernier et al., 2019; Lee et al., 2019), and, perhaps, memory biases. First, a link between insensitive care during infancy and memory for negative experiences was implied within an experiment examining whether or not preschoolers exhibited decreased (as opposed to maintained or enhanced) fear behavior across multiple encounters with a potentially frightening stimulus, which a friendly experimenter had told them was okay to touch (Tsotsi et al., 2018). Greater levels of insensitive care predicted the maintenance or enhancement of startle behavior across trials (Tsotsi et al., 2018). This finding is reminiscent of animal research wherein worse early life experience impaired neurophysiology associated with learning during neutral conditions but promoted learning during frightening ones (Bagot et al., 2009).

Second, in another examination of insensitive caregiving, Rifkin-Graboi et al. (2021) found no differences in the relation between insensitivity in infancy and preschoolers’ memory for associations between pictures of animals and stimuli that were not socioemotional in nature (i.e., food items) but a significant relation to items that were (i.e., pictures of school-aged children’s emotional faces), specifically in girls. These results were interpreted in light of conditional adaptation. Such an interpretation is also consistent with work linking enhanced early neighborhood violence and improved memory for dominance, but not aged based, relationships between people (Frankenhuis et al., 2020). It is also in keeping with findings indicating biases for angry faces and/or auditory information in physically abused children (Shackman et al., 2007), as well as a link between poverty exposure and better performance on an ecologically salient (e.g., pictures of money) working memory task but not on an abstract working memory task (Young et al., 2022). That is, the results were considered in reference to the idea that insensitive care may be a marker of environmental adversity (Perry et al., 2018). As such, children who have experienced comparatively more insensitive care may need to especially attend and remember (negative) social information, especially as they are more likely to have to independently navigate the social world at an earlier stage of development than their counterparts who have received more sensitive care (Rifkin-Graboi et al., 2019; Rifkin-Graboi et al., 2021; Rifkin-Graboi and Ngoh, 2022).

With such work in mind, it is possible that insensitive caregiving may foster the development of relational memory biases, in a conditionally adaptive manner with regards to some aspects of life, but at the same time leaving children vulnerable to the development of internalizing problems *via* the same mechanism. Nevertheless, before concluding that relational memory is a candidate process in the association between insensitive care and mood disorders, the link between insensitive caregiving and relational memory must be further explored and elaborated. First, to date, all the published research concerning insensitivity and relational memory has emanated from one cohort study, i.e., Growing Up in Singapore Towards Healthy Outcomes (GUSTO). Although the aforementioned GUSTO papers did not include entirely overlapping participants, testing the relation in a different sample is important for concerns regarding replication. In addition, as Rifkin-Graboi et al. (2021) included both happy and angry faces at encoding, and only happy faces at test, questions remain whether the prior findings specifically indicate a bias for negative, potentially threatening information, or a bias, for social information, in general.

Furthermore, the moderating role of gender requires consideration. As noted above, exploratory analyses within Rifkin-Graboi et al. (2021) indicate that a negative memory bias was apparent in preschool girls, but not boys. Although a variety of reasons could account for this finding, including sexual dimorphism in the pace of hippocampal development (Mu et al., 2020), given that, at least starting in the teenage years, girls may be more likely to suffer from depression (Kovacs et al., 2003), it is important to further examine gender as a possible moderator in the relation between early life care and relational memory.

Additionally, there are reasons to ask whether variation in maternal sensitivity, outside of infancy, has a similar impact on emotional relational memory, and supporting neuroanatomy at various stages of the life course. First, the timing of exposure to adversity has been found to have an impact on performance in tasks involving the hippocampus. For example, Dunn et al. (2016) found positive effects of abuse on memory prior to adolescence (i.e., early childhood sexual abuse positively relating to numerical working memory and late childhood physical abuse predicting better verbal word recall) but early adolescent sexual abuse to have a negative impact upon numerical working memory. Second, developmental stage may influence the ability to detect individual differences in forms of relational memory, namely relations between items (Item-Item), items and space (Item-Space), and the temporal sequence of items (Item-Time) (Lee et al., 2016).

The present study sought to explore influences and outcomes of emotional relational memory in the preschool years as it is an important area of investigation with regards to the emergence of mood disorders. To better understand the relationship between early life care, gender, and memory development, the current study examined predictions from maternal sensitivity when children were roughly four and a half years of age, and their relational memory roughly one year later. Relational memory was assessed for person-person (Item-Item), person-space (Item-Space), and person-sequence (Item-Time) relationships when separately viewing vignettes about happy and angry children. We hypothesized that early life insensitive caregiving would specifically predict better memory for angry, but not happy, stimuli.

In addition, in the context of memory development two additional research questions were examined in an exploratory capacity in a subset of children in our sample with available neuroimaging data. First, as the hippocampus is a key brain area involved in emotional memory (Strange et al., 2014; Lambert et al., 2017) and can be influenced by early life adversity (Lee et al., 2019), we asked whether hippocampal volume would associate with maternal sensitivity or aspects of relational memory that are influenced by maternal sensitivity (as indicated by the results of our main research question). Past work suggests that the impact of maternal sensitivity in infancy upon preschool hippocampal development is not bilaterally uniform (Lee et al., 2019), and hippocampal sub-regions may support different forms of relational memory at varying points in development (Guillery-Girard et al., 2013; Lambert et al., 2017). As such we focused on left and right hippocampal head, body, and tail volume. The analyses concerning hippocampal volume and sensitivity represent a subset of pre-registered analyses; these analyses examining hippocampal volume and relational memory are related (but not identical) to those laid out in the same pre-registration^1^. The difference is that in the current work our examination of the hippocampus is within the context of expanding upon relations between sensitivity and relational memory.

Second, to shed light upon the potential role of negative memory biases in the formation of risk toward depressive mood in the preschool years, we examined whether better memory towards angry, but not happy, stimuli associated with co-occurring maternally reported internalizing problems.

## 2. Materials and methods

The current work is part of a larger two-wave study, SPACE (Singapore Parenting and Cognition in Early Childhood) study, funded by The National Research Foundation (NRF2016-SOL001-003) and approved by the NTU-IRB (IRB Ref no. 2018-04-015). Relevant to the current study are data from the Wave One visit (i.e., maternal sensitivity data), Wave Two visit (i.e., child Relational Memory data), and the optional neuroimaging visit (i.e., child hippocampal volumetric data). Mothers and children respectively, provided informed consent and assent prior to participation.

Sixty-seven mother preschooler dyads were recruited *via* word-of-mouth, social media, the Science Centre Singapore, and *via* preschools/child-care centers for the Wave One visit, which occurred between February 2019 and January 2020, so prior to the first reported COVID case in Singapore. This visit took place in participants’ homes, except for one dyad who found it more convenient to come to the laboratory. Wave One visits included cognitive and/or academic game-like tasks for children, an interview, and questionnaires for the mothers, and, relevant to the current study, observational sessions that occurred about 1 h into (or slightly less than halfway through) the visit.

The Wave Two visit occurred an average of 418 (*SD* = 77) days (or roughly 1.14 years, *SD* = 0.21) after the first visit, between September-December 2020. Fifty-one of the Wave One participants attended these visits. Wave Two visits took place at the National Institute of Education’s Centre for Research in Child Development. This visit was similar to that conducted at age four and a half, but relevant to the current study, also included a Relational Memory task. During this visit children participated in seven tasks (one of which included training on a controller) plus the observational session. The first two tasks, outside the scope of the current study, assessed processing speed and children’s preference for caregivers. The other five tasks, including the Relational Memory task, were counterbalanced. Interspersed between the tasks was a play session wherein children practiced behaviors relevant to MRI (e.g., laying still) and a parent-child observational session.

The optional neuroimaging visit occurred between January-March 2021 and included 23 of the children taking part in the Wave One and Wave Two visits.

### 2.1. Participants

As noted above, there were 67 participants in the Wave One visit (relevant to maternal sensitivity) and 51 who returned for Wave Two (relevant to Relational Memory), though Wave One maternal sensitivity data was not available for two mother-daughter dyads due difficulties uploading to the server. Thus, 49 dyads contributed data to the maternal sensitivity and Relational Memory analyses, and all but one of these dyads also contributed internalizing data. Twenty-three children attended the neuroimaging session, but three did not enter the scanner and an additional two did not complete the MPRAGE scan required for volumetric analyses. Thus, eighteen children contributed usable neuroimaging data. The average age of the 49 children taking part in the majority of analyses was 4.5 years (*SD* = 0.28, Range = 4.02–4.99 years) at Wave One and 5.65 years (*SD* = 0.38, Range = 4.98–6.32) at Wave Two.

Maternal sensitivity at Wave One did not differ [*t* (63) = −0.495, *p* = 0.622] between the 47 dyads with Wave One maternal sensitivity data who also returned for Wave Two (*M* = 0.39, *SD* = 0.23) and the 16 dyads who did not return to the Wave Two visit and so did not have relational memory data (*M* = 0.42, *SD* = 0.19). Furthermore, when comparing the 49 children (23 girls and 26 boys) with the 18 (12 girls and 6 boys) for whom either maternal sensitivity or relational memory data were not available, the ratio of male and female children did not significantly differ from chance (*X*^2^ = 2.053, *p* = 0.152), nor did maternal ethnicity amongst those whose data were (44/49 ethnic Chinese) and were not (15/18 ethnic Chinese) included. Of the eight mothers who did not report being of Chinese ethnicity, two reported their ethnicity as Indian, four as Malay, and one as Javanese. For context, the majority of Singaporean citizens report Chinese ethnicity (> 75%). At Wave One all mothers but one reported being married and at Wave Two all mothers but two (including the aforementioned one) reported being married at Wave Two. All mothers reported that the Study Child lived with them at both visits. All mothers reported their levels of maternal education, except for one who did not return for the Wave Two visit. Ordinal level of maternal education did not significantly differ [*t* (64) = −0.607, *p* = 0.546] between those whose data were and were (*M* = 3.96, *SD* = 0.763) and were not (*M* = 3.82, *SD* = 0.883) included, with mothers reporting at least the completion of secondary education (“2”) and up to the completion of a postgraduate degree (e.g., M.A. or PhD., “5”).

The imaging subsample included nine girls and nine boys, with an average age of 6.01 years (*SD* = 0.36) and did not differ from the 49 other children who participated in the SPACE study in terms of ratio of male to female children (*X*^2^ = 0.049, *p* = 0.824), maternal Chinese ethnicity (*X*^2^ = 0.523, *p* = 0.470), or level of maternal education [*t* (64) = 1.278, *p* = 0.206].

### 2.2. Assessments

#### 2.2.1. Child gender

Male versus female status was recorded by the research assistant visiting the home during Wave One and reflects e.g., the family’s language usage when referring to the preschooler (e.g., “she”/“he”, “boy”/“girl”, “daughter”/“son”, “sister”/“brother,” etc.), the typical gender associated with the child’s name, and/or the way the child visually presented (e.g., wearing a dress, hair in bows, etc.). This definition does not consider whether children’s preferences, *per se*, were taken into account by their families, and, given local culture, likely also reflects sex assigned at birth.

#### 2.2.2. Maternal sensitivity

##### 2.2.2.1. Observational sessions

Maternal sensitivity was coded from video records of the observational sessions, which lasted a total of 15 min and consisted of four different scenarios: (1) free play/arts and crafts; (2) clean up; (3) math game; (4) novelty, in the form of an experimenter wearing a mask, with the option of then playing with the masks. Scenarios were counterbalanced across participants, with the exception that clean-up, by necessity, always immediately followed free play/arts and crafts. Each scenario was approximately 3 min and 45 s long, with the last 45 s constructed to elicit divided attention on the part of the mother. During the last 45 s of each scenario mothers were given an iPad including close captioned instructions for the next scenario or, in the case of the last scenario, a short wrap up video. An instructional video was also given to the mothers before the start of the first scenario.

All instructional videos provided some suggestions of what dyads could do during the scenario, but purposefully left it to the mothers to convey the task’s implicit goals, and to decide the extent to which she wanted to manage/comply. Each instructional video ended with the phrase, “During this activity please behave as you normally would in a similar situation.” However, in the clean-up session, additional instructions were given, i.e., “During clean-up please behave as you normally would in a similar situation. You may help your child, however, please have your child do at least 75% of the cleaning up.”

##### 2.2.2.2. Scoring the observations: The preschool MBQS

The video records were coded using the *Maternal Behavior Q Sort Adapted for Preschool (Preschool MBQS)* (Pederson et al., 2013). The Preschool MBQS contains 25 items that are sorted into one of 5 categories. The category labels range from “least like the observed interaction” to “most like the observed interaction,” and are associated with a score ranging from “−2” to “+2.” Each category is permitted to have exactly five items. Thus, when the coder has finished sorting the items, there should be an equal number of cards that have received a score of “−2”, “−1”, “0”, “+1”, or “+2.” This then allows for the scores of the 25 items to be correlated against standard scores that have been assigned to each item by the system’s developers. Scores assigned by the system’s developers reflect the ways in which the cards would be sorted if describing a prototypically sensitive interaction. Ultimately then, each observed interaction session receives a summary score (i.e., its correlation with the prototypically sensitive interaction) ranging from −1 to +1, with −1 indicating an interaction that is the most dissimilar to the hypothetical prototypically sensitive interaction and +1 indicating an interaction that is identical to the hypothetical prototypically sensitive interaction. In the current work, we created an average score, per dyad, based on the −1 to +1 scores assigned to each of the four sessions in which the dyad participated.

##### 2.2.2.3. Training to code the preschool MBQS

Data were coded by two Southeast Asian coders, Lit Wee Sim (hereafter “LWS”, the current paper’s fifth author), and Clara Tan (CT). LWS had previously been trained in the conceptually and operationally similar MBQS by two of its developers (S. Bento and D. Pederson) and at the time of coding the current sample had already scored hundreds of video records using the mini-for-video MBQS for a separate large Singaporean study. At the time of coding, LWS had also achieved reliability (*via* Minnesota) in the organized categories of the strange situation, and so had a good foundation in concepts related to sensitivity and its impact on attachment relationships. To prepare for the current study, LWS, the lead author [ARG, also trained by S. Bento and D. Pederson on the infancy versions of the MBQS as well as Main and Hesse’s Frightening/Frightened (FR) system, Hesse and Main (2006)], and another local coder (Shamini Sanmugam, SS, with a similar background to LWS) discussed the preschool MBQS items and evaluated pilot scenarios that roughly followed the same procedure as those employed for the current study. Pilot scenarios were similar to those included here for Math, Novelty, Free Play, and Clean-Up, but, during the pilot, a separate scenario was designed for Divided Attention; in addition, during piloting two other scenarios (i.e., storybook reading and a language game) not included in the current study were also conducted. LWS and SS independently evaluated a number of pilot scenarios (including 26 pilot scenarios most similar to those used here) and reached consensus on any notable discrepancies. LWS then independently coded remaining pilot cases. Pilot cases, primarily those coded by both LWS and SS, were then used for CT’s training. Prior to scoring CT had also worked with ARG as part of the “Undergraduate Research Experience on Campus,” and during this time had been introduced to readings and concepts relevant to Attachment Theory and sensitivity.

##### 2.2.2.4. MBQS reliability

Following training, LWS and CT coded data relevant to the larger study from which the current data is part. Approximately twenty percent of all available data in the larger data set (i.e., a total of 13 cases per each of the four task scenarios, including data from 35/67 children) were double coded. For these overlapping cases, one coder was pre-assigned as the primary coder and the other as the reliability coder and for those jointly coded cases, the pre-assigned primary coder’s score was entered into the database, except in cases where there were notable discrepancies between the coders (i.e., > 0.25 difference in MBQS sensitivity score). In such cases the coders met to discuss, and a finalized consensus score was used. In total, the current paper’s data set used individual participant averages based upon scores, from, respectively, LWS, consensus, and CT as follows: clean-up: 22,1,26; mask: 22,2,25; math: 26,3,20; free play: 27,5,17). Inter-rater reliability was assessed through two-way mixed model absolute intra-class correlation coefficients, which indicated reliability of 0.866 across these 52 scenarios (95% CI: 0.767 to 0.923). According to guidelines, this indicates “good” reliability, as less than 0.5 is thought to indicate poor reliability; 0.5 to 0.75 moderate; 0.75 to 0.9 good; and above 0.9 excellent (Koo and Li, 2016).

#### 2.2.3. Relational memory task

The Relational Memory task was conducted on a touchscreen laptop. Experimenters told children that they would be told a story about similarly aged children who “go to school” [i.e., the Target (T) characters] and are allowed to play with other children [i.e., the Friend (F) characters] on a big field at break time. The pictures used in the story (experiment) were taken from the Child Affective Emotional Faces (CAFÉ’) Data Bry database (LoBue and Thrasher, 2015). The Café stimuli included a collection of photographs taken of 2- to 8-year-old children asked to depict angry or happy expressions, chosen to, as much as possible, represent the three majority races present in Singapore (Malay, Indian, and Chinese). Previously 100 adults had rated the pictures to confirm emotional expressions (Happy *M* = 90.92, *SD* = 5.33; Angry *M* = 75.75; *SD* = 9.57). Stimuli faces in this study include six female and six male models (LoBue and Thrasher, 2015).

**FIGURE 1:**
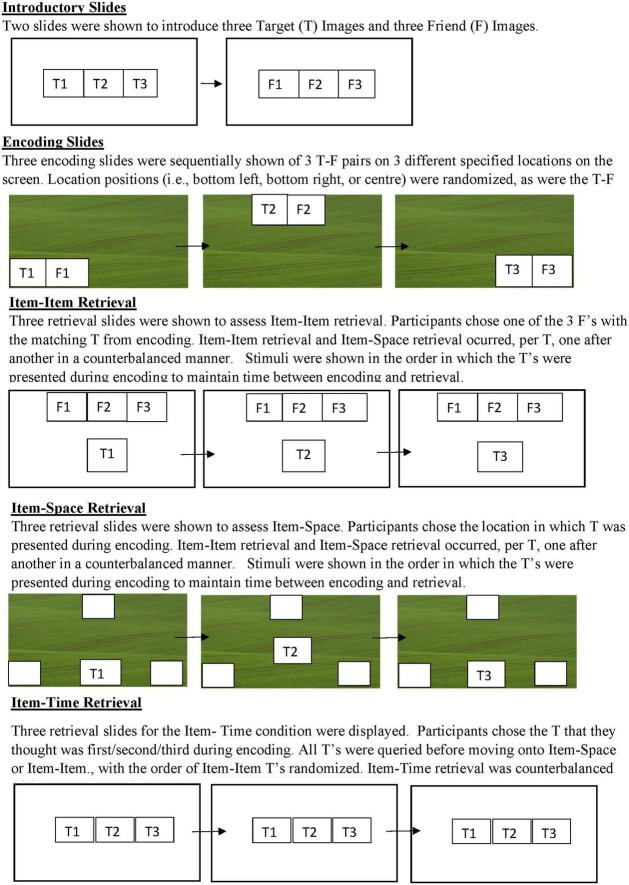
Graphic depiction of the task order within an Emotional Block. Happy and Angry blocks were counterbalanced within the participants. T (Target) and F (Friend) identities were randomly chosen across blocks, such that there was never the same T or F displayed in the Angry and Happy conditions. The usage of 1-2-3 is for heuristic purposes only. The order and pairings of T’s and F’s was random with no repetition across emotional blocks. T and F pictures were chosen from the NIMH ChEFS picture bank (Egger et al., 2011).

This task had two counterbalanced emotional blocks depicting children with emotional expressions: Happy and Angry. Stimuli included in one block was not reused in the next. Otherwise, the procedure across the emotional blocks was similar. Both emotional blocks had three encoding slides followed by the test slides. Both conditions included encoding and test slides relevant to the three memory conditions (i.e., Item-Item, Item-Time, and Item-Space, see Figure 1).

In each emotional block, study children were exposed to two introductory slides, three encoding slides, and nine retrieval slides. In the first introductory slide, participants were introduced to pictures representing the three target (T) characters. In the second introductory slide, participants were introduced to pictures of three of T’s friends (F). To be inclusive of all genders, but to also help children further identify with the task, children presenting as female were given a version wherein the T’s were of girls and the F’s were of boys, whereas children presenting as male were given a version where the T’s were of boys and the F’s were of girls.

During the encoding slides, participants were sequentially shown different T-F pairs one-at-a-time at one of three locations (bottom left, bottom right, and top right) on a picture of a green field. The T-F pairings, locations, and order of presentation was randomized. Participants were asked to pay attention to *where* (Item-Space) on the field T went; *whom* (Item-Item) of three F’s went with T; and in relation to other T’s, *when* (Item-Time) T went to the field (i.e., first, second, or third). When the pairs were introduced the experimenter would say, “(First/Next/Lastly), we have this (Boy/Girl) who went to play with this (Girl/Boy)! And they had a (great time/huge fight),” with “great time” versus “huge fight” varying according to the Emotional Block. To ensure that children were attending to the relevant information, upon presentation the experimenter asked the child to point to the place (Item-Space) that both the T and F (Item-Item) were located and used a word-cue like first, second, or third (Item-Time) when doing so. In cases where the child did not point to a pair, before advancing to the next encoding slide, the experimenter would give an additional prompt.

Then, before retrieval children were given a preparatory slide that displayed the three *T’*s and were told, “Now that I finished the story, do you remember these boys/girls? I am going to be asking you some questions about them and I want you to touch the screen where you think the answer is!” They were then asked three questions (three times for each target stimuli) relevant to each of the memory conditions (i.e., Item-Item, Item-Space, and Item-Time). For Item-Item one T was shown on a slide with all three F’s, and children were asked “Can you tell me which (Boy/Girl) this (Girl/Boy) went to the field with?” This was repeated for all three T’s. For Item-Space, children were shown a T on the green field with tan boxes outlined in the three relevant locations and asked, “Can you tell me where on the field he/she went to play?” This was repeated for all three T’s. For Item-Time, children were shown all three T’s on one slide and asked, “Can you tell me which (Boy/Girl) went to the field (first/second/third)?.” This was repeated three times for all three *T*’s. Across conditions if children did not respond they were encouraged to just try their best.

The number of correct responses per emotional (Happy and Angry) and memory (Item-Space, Item-Item, and Item-Time) condition was recorded *via* the Eprime software, yielding six scores (e.g., Happy Item-Item; Angry Item-Item, Happy Item-Space, etc.), ranging from 0 to 3 per scale.

#### 2.2.4. Internalizing problems

Mothers completed the Strength and Difficulties Questionnaire (SDQ, Goodman, 1997) at the Wave Two visit wherein they rated whether items accurately described their child’s behavior in a 3-point scale (0 = not true, 1 = somewhat true, 2 = certainly true). Based on the original scoring, an Internalizing Problems score can be calculated by summing up the item responses in two of the questionnaire’s five subscales, i.e., emotional problems and peer problems, leading to a maximum score of 20. In line with past Singaporean research (Bull et al., 2016) an Internalizing score was created that differed from the scale score defined by Goodman and colleagues (Goodman and Goodman, 2009) by omitting three items (Peer Relationship Items 11, 14, and 23) from its calculation, leading to a potential maximum score of 14. Internal reliability using all available data (*n* = 50) from the Wave Two time point was α = 0.78 (within this same sample, the internal reliability for the Internalizing score based on the Goodman et al. (Goodman and Goodman, 2009) criteria was α = 0.70).

#### 2.2.5. Neuroimaging

MRI data were acquired using a Siemens (Siemens Healthineers, Erlangen, Germany) Prisma 3T system using the Q-body coil for signal transmission with reception on a 32-channel head coil. A T_1_-weighted Magnetization PRepAred Gradient Echo (MPRAGE) with 1 mm^3^ isotropic voxels, 192 × 192 × 192 matrix, TI/TE/TR = 877/2.08/2,000 ms was collected for volume-based morphometry (VBM) and was repeated if significant motion was detected on acquisition (based on recommendation of experienced radiographer).

MPRAGE data were processed using the Freesurfer (v7.1.1, MGH, Harvard) image analysis suite consisting of removal of non-brain tissue (Ségonne et al., 2004), Talairach transformation, intensity normalization (Sled et al., 1998), and segmentation (Fischl et al., 2002, 2004a,b). Data were further processed to extract hippocampal and amygdala subfields using the automated pipeline in Freesurfer (Iglesias et al., 2015).

### 2.3. Statistical analyses

Prior to assessing relations of interest, Spearman correlations were conducted to determine whether to control for maternal education and child age (i.e., relevant testing date minus birth date, divided by 365, with testing date corresponding to Wave One, Wave Two, and at Neuroimaging, as appropriate). If associations between these variables and both variables of interest (e.g., the predictor and outcome) were found greater than *p* > 0.05, covariates were to be entered into the models. Gender was similarly examined, *via* a Student’s *T*-Test, as a potential covariate for the neuroimaging and internalizing symptoms analyses.

#### 2.3.1. Relational memory and maternal sensitivity

Following this, a mixed ANCOVA was performed *via* IBM SPSS Statistics Version 28 (SPSS Inc., Chicago, IL, USA). The between-subject variables were gender and maternal sensitivity (entered as a covariate due to software specifications) and the within-subject variables were the two-level emotion variable (i.e., Happy and Angry) and the three-level memory condition variable (i.e., Item, Time, and Space). Any significant main effects for within subject variables were subsequently probed with pairwise comparisons. Significant interactions involving the between-subject variables were probed *via* regression (see Section “3. Results” for additional details). The criterion for statistical significance for follow-up analyses was > 0.05.

#### 2.3.2. Relational memory and hippocampal volume

Spearman Correlations were conducted to determine whether whole brain corrected hippocampal subregion volumes associated with (a) aspects of relational memory predicted by maternal sensitivity as observed above and (b) maternal sensitivity during Wave One. The criterion for statistical significance was set to > 0.008 (i.e., 0.05 divided by 6) to account for the fact that six regions were considered. A formal test of mediation was not considered given that the acquisition of neuroimaging data did not precede that of relational memory data.

#### 2.3.3. Relational memory and internalizing problems

Spearman correlations were used to examine the relation between internalizing problems and any forms of (a) aspects of relational memory predicted by maternal sensitivity as observed above (2.3.1) (b) maternal sensitivity during Wave One A formal test of mediation was not considered given that the acquisition of neuroimaging data did not precede that of internalizing data.

## 3. Results

Maternal sensitivity was not related to whether children took part in the Happy or Angry emotional condition first. In addition, in no case was child age or maternal level of education related to both variables of interest. Thus, our models did not include these variables as covariates. Likewise, gender was not related to hippocampal subregion volumes or internalizing problems and so was not included in those analyses.

Descriptives for maternal sensitivity, relational memory, hippocampal subregion volumes, and internalizing problems can be found in Table 1.

**TABLE 1:** Descriptive statistics for variables of interest.

	Maternal sensitivity	Happy item- time	Happy item- item	Happy item- space	Angry item- time	Angry item- item	Angry item- space	Right hippocampal tail (corrected)	Left hippocampal tail (corrected)	Right hippocampal body (corrected)	Left hippocampal body (corrected)	Right hippocampal head (corrected)	Left hippocampal head (corrected)	SDQ internalizing (local factor)
Mean	0.39	2.00	1.51	1.10	1.67	1.65	1.33	551.93 (3.38E-04)	530.14 (3.68E-04)	1144.45 (7.91E-04)	1125.39 (7.78E-04)	1658.40 (1.15E-03)	1582.31 (1.09E-03)	2.06
Std. deviation	0.23	1.17	1.00	1.07	1.23	1.13	0.99	43.21 (4.15E-05)	42.08 (4.22E-05)	92.57 (5.31E-05)	102.59 (6.70E-05)	113.73 (6.21E-05)	126.57 (8.20E-05)	2.44
Minimum	–0.10	0	0	0	0	0	0	480.67 (3.29E-04)	445.47 (2.89E-04)	959.60 (6.88E-04)	980.33 (6.36E-04)	1417.55 (1.04E-03)	1393.73 (9.05E-04)	0
Maximum	0.68	3	3	3	3	3	3	629.72 (4.48E-04)	598.37 (4.23E-04)	1396.24 (8.76E-04)	1360.97 (8.78E-04)	1940.85 (1.25E-03)	1958.38 (1.22E-03)	8
Sample size	49	49	49	49	49	49	49	18	18	18	18	18	18	48

Maternal sensitivity was collected at Wave One. Relational memory and internalizing problems were collected at Wave Two. Hippocampal volumes were collected at a neuroimaging visit scheduled after the Wave Two visit. Corrected values are within parentheses.

### 3.1. Overall effects of gender, maternal sensitivity, emotion condition, and memory condition

Shapiro-Wilkes tests indicated that neither memory nor maternal sensitivity were normally distributed (*p* < 0.05), and that there were two outliers in the data (i.e., for girls within the Happy-Space condition), as assessed by inspection of a boxplot for values greater than 1.5 box-lengths from the edge of the box. In one case a review of the case notes suggested that the child may not have understood the task. Thus, analyses were conducted with and without both outlying cases. Because the pattern of results remained similar in both analyses (i.e., there were no changes in overall effects with regards to their statistical significance, *p* < 0.05, and in only one instance was there a difference in the pairwise comparisons) we report results with these two cases included.

There was homogeneity of variances, as assessed by Levene’s test for equality of variances (*p* > 0.05). Mauchly’s test of sphericity indicated that the assumption of sphericity was not met with regards to the memory condition χ2 (2) = 10.733, *p* = 0.005, but was met with regards to the interaction between emotion and memory conditions, χ2 (2) = 0.947, *p* = 0.623. With regards to the between subject variables, a significant main effect of sensitivity [*F* (1,45) = 4.241, *p* = 0.45, η_*p*_^2^ = 0.086] was observed with no significant main effects of gender [*F* (1,45) = 0.147, *p* = 0.703, η_*p*_^2^ = 0.003], nor the interaction between gender and sensitivity [*F* (1,45) = 0.006, *p* = 0.941, η_*p*_^2^ = 0.000]. As detailed in Table 2, with regards to the within-subject variables, there were no significant effects of emotion condition or memory condition. However, a significant interaction was observed between emotion and memory condition as well as between emotion condition, memory condition, and maternal sensitivity.

**TABLE 2:** Within-subject effects of emotion, memory type, and their interactions.

Effect	Df	F	Sig.	Partial eta squared
Emotion	1,45	0.426	0.517	0.009
Emotion × Sensitivity	1,45	0.343	0.561	0.008
Emotion × Gender	1,45	0.467	0.498	0.010
Emotion × Gender × Sensitivity	1,45	1.718	0.197	0.037
Memory	1.644, 73.986	1.461	0.239	0.031
Memory × Sensitivity	1.644, 73.986	0.502	0.571	0.011
Memory × Gender	1.644, 73.986	0.368	0.652	0.008
Memory × Gender × Sensitivity	1.644, 73.986	0.570	0.535	0.013
Emotion × Memory	2, 90	6.004	**0.004**	0.118
Emotion Memory × Sensitivity	2, 90	5.067	**0.008**	0.101
Emotion × Memory × Gender	2, 90	1.444	0.241	0.031
Emotion × Memory × Gender × Sensitivity	2, 90	3.052	0.052	0.064

As the assumptions concerning sphericity were violated for the Memory condition analyses, a Greenhouse-geisser correction was applied when examining Memory, Memory × Sensitivity, Memory × Gender, and Memory × Gender × Sensitivity. Bold values represent the statistically significant (*p* < 0.05).

Pairwise comparisons were based on the estimated marginal means with no adjustment. Pairwise comparisons considering Emotion differences within each Memory condition did not reveal any significant effects. However, pairwise comparisons considering Memory type within each Emotion condition indicated that within the Happy emotional condition, memory accuracy within the Time condition was significantly better than memory within the Item condition (Mean Difference = 0.527, se = 0.210, *p* = 0.016) or the Space condition (Mean Difference = 0.913, se = 0.224, *p* < 0.001), and that memory accuracy within the Item condition was significantly better than memory within the Space condition (Mean Difference = 0.387, se = 0.178, *p* = 0.035). Within the Angry condition, only one significant difference was observed, namely that accuracy for Item relationships was better than accuracy for Space relationships (Mean Difference = 0.386, se = 0.172, *p* = 0.030). However, when conducted without the two outlying cases, we also observed that children performed significantly better in the Angry Time condition than the Angry Space condition.

Despite the fact that the two-way interaction indicated no emotion differences within each memory type, we chose to probe our three-way interaction between Memory condition, Emotion condition, and sensitivity by calculating scores for the Happy and Angry conditions separately within each memory condition (e.g., Happy Space minus Angry Space), rather than by calculating scores for the varying Memory types within each Emotion condition. This choice was based upon the hypotheses of interest for this paper- namely that sensitivity may bias memory according to its emotional content. Difference scores were used as outcome variables. Regressions were bootstrapped at 10,000 simulations and indicated that only the association between maternal sensitivity and the Happy-Angry difference score within the Space condition reached significance [*B* = 2.451, se = 0.969, *p* = 0.014, 95% CI (0.572, 4.340)]. Differences scores in Item-Item [*B* = −0.605, se = 0.995, *p* = 0.546, 95% CI (−2.472, 1.415)] and Item-Time [*B* = −0.542, se = 0.945, *p* = 0.563, 95% CI (−2.379, 1.340)] were not significant.

**FIGURE 2:**
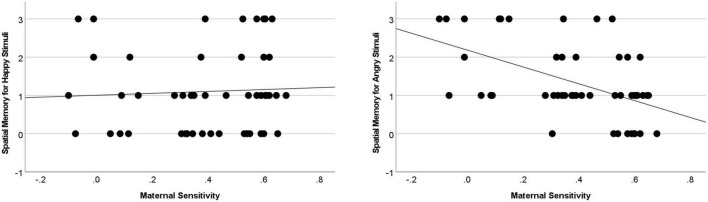
Insensitive caregiving predicted the difference between Angry and Happy memory during the item-space condition (*B* = 2.451, se = 0.969, *p* = 0.014, 95% CI [0.572, 4.340]), as well as memory for Angry (but not Happy) items [*B* = –2.203, se = 0.551, *p* < 0.001, 95% CI (–3.264,–1.094)].

Following this, to more comprehensively understand the direction of effects, two additional bootstrapped regressions were conducted with maternal sensitivity as the predictor and child memory for item-space associations in the happy, and separately, angry conditions. As indicated in Figure 2, there was a significant (*p* < 0.008) negative association between sensitivity and relational memory in the Angry Item-Space condition [*B* = −2.203, se = 0.551, *p* < 0.001, 95% CI (−3.264,−1.094)]. Maternal sensitivity did not significantly predict memory in the Happy Item-Space condition: (*B* = 0.249, se = 0.764, *p* = 0.743, 95% CI [−1.176, 1.828]).

### 3.2. Hippocampal volume

Hippocampal data were normally distributed; however, in this subsample maternal sensitivity and the difference between Item-Space performance in Happy and Angry conditions, as well as within the Angry condition itself exhibited non-normal distributions. Hence, the Spearman Rho test was used to examine relationships.

As reported in Table 3, none of the hippocampal subregion volumes significantly associated with maternal sensitivity.

**TABLE 3:** Spearman Rho correlations between hippocampal subregion volume, maternal sensitivity, and relational memory in the space condition (i.e., Happy versus Angry and Angry).

Hippocampal subregion	Maternal sensitivity	Angry space	Angry versus happy space
Left head	-0.180	0.423	-0.207
Right head	-0.262	0.022	-0.114
Left body	-0.015	0.531*	0.242
Right body	0.007	0.639**	-0.509*
Left tail	-0.363	0.230	0.027
Right tail	-0.196	0.570*	-0.178

Hippocampal volume was corrected for whole brain volume. Significance values, *p < 0.05; **p < 0.01 (uncorrected).

With regards to relationships between hippocampal volumes and relational memory, we focused upon potential associations between hippocampal subregions and the difference between Happy and Angry Item-Space relational memory, as well as variation within Angry Item-Space relational memory. We focused our analyses on these aspects because these were the only forms of memory predicted by maternal sensitivity (see Section “3.2. Hippocampal volume”).

**FIGURE 3:**
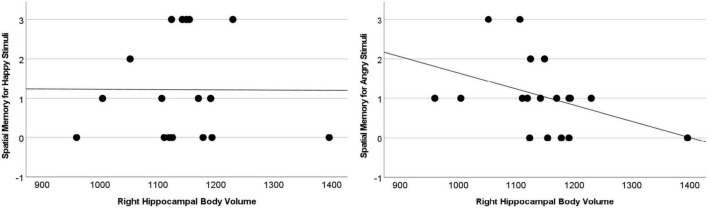
Memory for the difference between Angry and Happy stimuli in the space condition associated with larger right hippocampal body volumes (Rho = 0.639, *p* = 0.004).

The difference between Angry and Happy stimuli in the Space condition revealed an association with the bilateral hippocampal body [Spearman Rho: 0.531, *p* = 0.023 (left); 0.639, *p* = 0.004 (right)], extending in the right hemisphere to the tail (Rho = 0.570, *p* = 0.013). Still after correction for multiple comparison only the association with the right hippocampal body achieved significance at *p* < 0.008 (see Figure 3).

Besides the difference scores, the substantial majority of analyses examining the association between the hippocampus (head, body, tail separately for the right and left hemispheres) and relational memory for Angry Item-Space suggested a negative association. Yet, none of the correlations remained significant after Bonferroni correction, and only one was significant prior to Bonferroni correction (i.e., uncorrected Rho = −0.509, *p* = 0.031 for the association between right body and relational memory in the Angry Item-Space condition).

### 3.3. Internalizing problems, relational memory, and maternal insensitivity

Spearman Rho’s were used to assess relations as none of the variables of interest were normally distributed. Forty-eight children were examined in these analyses.

Internalizing problems were not associated with the relational memory difference score between Angry versus Happy stimuli in the Space condition (Rho = 0.194, *p* = 0.187), nor with relational memory in the Angry Item-Space condition (Rho = −0.102, *p* = 0.489). Neither did they associate with maternal sensitivity (Rho = −0.132, *p* = 0.370).

## 4. Discussion

Here we investigated links between maternal sensitivity, emotional memory biases, and hippocampal subregions in preschoolers to further elucidate the neurobiology linking variation in early life care to psychological risk. Consistent with theory and research suggesting that environmental exposures influence the development of memory and schemas important to close relationships and risk management (Main, 1981; Belsky et al., 1991; Vandevivere et al., 2014; Biro et al., 2015; Peltola et al., 2015; Bosmans et al., 2020), here we found that comparatively lower levels of maternal sensitivity when children were four and a half years of age predicted better relational memory when angry, as opposed to happy, stimuli were involved. Considering the results from analyses examining performance within the emotional conditions, this difference was likely due to a boost in memory performance with angry stimuli. In addition, in a small (*n* = 18) subset of children we found an association between hippocampal subregions and the difference in performance for angry versus happy relational memory. As such, the current work adds to an increasing number of papers linking early life environmental exposures to memory biases (Rifkin-Graboi et al., 2021) as well as hippocampal development (Rao et al., 2010; Luby et al., 2012, 2013, 2016; Rifkin-Graboi et al., 2015; Bernier et al., 2019; Lee et al., 2019; Wang et al., 2019), potentially important to the development of children’s adaptive functioning and psychological health.

Still, despite the associations between maternal sensitivity, memory, and hippocampal development, associations with internalizing behavior were not significant. Nevertheless, although, at a population health level, maternal sensitivity may be important given its likely pervasiveness (De Wolff and Van Ijzendoorn, 1997; Moran et al., 2008), published meta-analytic effect sizes for the link between maternal sensitivity and internalizing behavior are rather weak *r* = −0.08, and known to be moderated by SES groups, with lower SES samples showing larger effects, *r* = −0.14 (Cooke et al., 2022). Therefore, our results with a small sample of highly educated Singaporean dyads may be expectable. In fact, according to University of California at San Francisco’s (Hulley et al., 2013) a sample size of 1,224 would be necessary for an analysis with 80% power. Indeed, our effect of Rho = −0.132 is similar to the meta-analytically reported 95% CI’s (i.e., −0.12 to −0.05) (Cooke et al., 2022). As such, future research may wish to extend the current findings in a larger higher risk group, within the context of a Three Wave Study where a formal test of mediation is feasible. In addition, future research may wish to examine whether genetic risk toward internalizing difficulties (Asarnow et al., 2014) and/or other forms of prior and concurrent experience moderate potential links between sensitivity, relational memory, and internalizing problems. It is easy to imagine that negative memory biases could be beneficial in some circumstances, perhaps preventing the escalation of interpersonal difficulties and facilitating socially adaptive behavior during encounters with others who have also experienced insensitive care, but harmful when the majority of peers have had more sensitive caregiving experiences, or with regards to other domains. Indeed, one Singaporean study reported relatively low rates of sensitive caregiving, as well as an inverse relation between aspects of sensitive care and female peer likability, despite some positive associations with components of language development (Cheung, 2021).

In the current work we only considered one potential moderator—gender (likely identical to sex assigned at birth for this sample) and did not find any main or interaction effects. Although we had anticipated that associations would be stronger in females than males, this lack of interaction may not be surprising as past work indicates that male-female differences in depression may not be apparent until the adolescent years (Kovacs et al., 2003). Thus, the current findings may be best interpreted within the developmental context in which they were assessed.

Indeed, the developmental stage at the age of relational memory assessment may have played a substantial role in the specifics of the observed associations, both in terms of memory form (e.g., Item-Space), and hippocampal subregion (e.g., right posterior). First, it is notable that we specifically found relations between sensitivity and a preference for angry over happy memory when assessing memory for where items were in relation to space (Item-Space), but not in relation to one another (Item-Item, Item-Time). Indeed, past developmental research (Lee et al., 2016) implies it may be easiest to observe differences in Item-Space memory before the age of 10. In Lee at al’s (2016) study of differences between adults’ and children’s Item-Space, Item-Time, and Item-Item memory they report that between the ages of eight to eleven children showed the best memory for Item-Space associations, followed by Item-Time, and then Item-Item associations (also see Lee et al., 2020); and that Item-Item memory may not be better than chance before age ten. In addition, in combination with work suggesting that insensitive caregiving may speed up hippocampal and memory development (Rifkin-Graboi et al., 2015; Rifkin-Graboi et al., 2018; Bernier et al., 2019) other work (Guillery-Girard et al., 2013) could suggest that Item-Space relational memory, unlike Item-Item and Item-Time memory, may be a good candidate for individual difference research during childhood given its linear development. That is, in the case of Item-Space memory, insensitivity could be expected to both speed up memory capability and bias memory toward negative emotions. In contrast, since Item-Item memory does not exhibit linear development across ages 6 to 10, the effects of insensitivity may work at odds with one another masking results; and Item-Time memory may not show sufficient variance to detect emotion related differences, given that it is relatively stable from six to nine before showing improvement (Guillery-Girard et al., 2013). Still, this interpretation is speculative, given that our planned comparisons did not find Memory condition effects, and pairwise comparisons within each emotion suggested that on our task preschoolers exhibited the worst memory for Item-Space associations.

In addition, the developmental stage may have influenced which hippocampal subregions were most related to emotion related differences in spatial relational memory. Though past work suggests that the anterior hippocampus may play an important role in emotional memory (Fanselow and Dong, 2010), an examination of 8–10-year-olds found the posterior hippocampus is specifically activated during the encoding of angry (but not happy) face-context associations and that its activity associates with greater accuracy in recalling these associations (Lambert et al., 2017).

Finally, by investigating maternal sensitivity in the preschool years, in combination with similar research examining maternal sensitivity’s prediction to relational memory in preschool (Rifkin-Graboi et al., 2021), the current work contributes to studies seeking a better understanding of the ways in which early life experience may contribute to memory development (Dunn et al., 2016) and suggests that insensitive care’s role on memory biases may be similar regardless of time point of early life exposure. Supporting this idea, differences in the way parents discuss autobiographical memory, including the identification of positive and negative events, predicts preschoolers’ accurate autobiographical memory of discussed events (e.g., Lawson et al., 2021). Nevertheless, given that variation in maternal sensitivity is likely somewhat stable in low-risk groups (Behrens et al., 2012), it remains possible that the current effects were driven by earlier exposure to insensitive care.

In sum, the current research further supports the idea that early life caregiving adversity may influence negative memory biases. In addition to replication in a substantially larger sample, future research may wish to examine associations at particular stages in the memory process. Prior work suggests that variation in caregiving experience could impact attentional processes at the time of encoding (Shackman et al., 2007). However, it is also possible that variation in exposure to insensitive care also biases storage and/or retrieval. For example, insensitivity is linked differences in basal cortisol levels (Berry et al., 2017), and cortisol can influence storage (Bisaz et al., 2014) and retrieval (Schilling et al., 2013), as well as biases toward emotional and/or negative information at retrieval (Mickley et al., 2017).

In addition, an important next step will be to determine the functional significance of such biases within varying caregiving contexts, as this could have important implications for prevention programs. Attachment theorists have long hypothesized that relationships are guided by cognitive-affective models, built up from experience, that guide behavior in conditionally adaptive ways (Main, 1981; Belsky et al., 1991). Likewise, there is evidence that forms of neurodevelopment, associated with adversity, can benefit individuals within certain contexts of risk (Gee et al., 2013; Ellis et al., 2017; Frankenhuis and Amir, 2022). If future research were to find that amongst children experiencing insensitive care, those who develop negative biases fare better in their family relationships then the implication might be that that prevention programs should not attempt to change negative biases without first changing caregiver sensitivity. Similarly, targeting biases could be problematic if future research were to find that children with negative memory biases are more socially adept than those without such biases specifically when they live in communities where insensitive care is the norm (but not when they live in communities where sensitive care is the norm). However, if biases are not found predictive of relatively better outcomes and are associated with future difficulties, then in the future it could be important to consider interventions targeting children themselves before problems occur.

## Data availability statement

Much of the original contributions presented in this study are publicly available. This data can be found here: https://researchdata.nie.edu.sg/dataset.xhtml?persistentId=doi:10.25340/R4/FIJDCJ. While certain forms of raw data (e.g., video files) cannot be shared for reasons of participant confidentiality, all reasonable requests for supporting de-identified data that do not impinge upon confidentiality will be honored.

## Ethics statement

The studies involving human participants were reviewed and approved by Nanyang Technological University NTU-IRB (IRB Ref no. 2018-04-015). Mothers and children respectively provided written informed consent and assent prior to participation.

## Author contributions

AR-G, ST, and KL conceived and designed multiple aspects of the study, with AR-G writing and performing the analyses herein, and ST and KL critically reviewing and editing the manuscript. LWS, NS, and MS contributed primarily to the conception, preliminary analysis, and writing of methods related to, respectively, maternal sensitivity, relational memory, and neuroimaging. All authors contributed to manuscript revision, read, and approved the submitted version.

## Footnotes

1
https://osf.io/4xtf5

## References

[B1] Ainsworth M. D. (1969). Maternal sensitivity scales. *Power* 6 1379–1388.

[B2] Asarnow L. D. Thompson R. J. Joormann J. Gotlib I. H. (2014). Children at risk for depression: memory biases, self-schemas, and genotypic variation. *J. Affect. Disord.* 159 66–72. 10.1016/j.jad.2014.02.020 24679392 PMC4000236

[B3] Bagot R. C. Van Hasselt F. N. Champagne D. L. Meaney M. J. Krugers H. J. Joels M. (2009). Maternal care determines rapid effects of stress mediators on synaptic plasticity in adult rat hippocampal dentate gyrus. *Neurobiol. Learn. Mem.* 92 292–300. 10.1016/j.nlm.2009.03.004 19292996

[B4] Behrens K. Y. Hart S. L. Parker A. C. (2012). Stability of maternal sensitivity across time and contexts with Q-sort measures. *Infant Child Dev.* 21 348–355.

[B5] Belsky J. Steinberg L. Draper P. (1991). Childhood experience, interpersonal development, and reproductive strategy: An evolutionary theory of socialization. *Child Dev.* 62 647–670. 10.1111/j.1467-8624.1991.tb01558.x 1935336

[B6] Bernier A. Dégeilh F. Leblanc É Daneault V. Bailey H. N. Beauchamp M. H. (2019). Mother–Infant Interaction and Child Brain Morphology: A Multidimensional Approach to Maternal Sensitivity. *Infancy* 24 120–138. 10.1111/infa.12270 32677197

[B7] Berry D. Blair C. Willoughby M. Granger D. A. Mills-Koonce W. R. Family Life Project Key Investigators. (2017). Maternal sensitivity and adrenocortical functioning across infancy and toddlerhood: Physiological adaptation to context? *Dev. Psychopathol.* 29 303–317. 10.1017/S0954579416000158 27065311 PMC5777168

[B8] Biro S. Alink L. R. A. Huffmeijer R. Bakermans-Kranenburg M. J. IJzendoorn M. H. V. (2015). Attachment and maternal sensitivity are related to infants’ monitoring of animated social interactions. *Brain Behav.* 5:e00410. 10.1002/brb3.410 26807337 PMC4714637

[B9] Bisaz R. Travaglia A. Alberini C. M. (2014). The neurobiological bases of memory formation: from physiological conditions to psychopathology. *Psychopathology* 47 347–356. 10.1159/000363702 25301080 PMC4246028

[B10] Bosmans G. Bakermans-Kranenburg M. J. Vervliet B. Verhees M. Van I. M. H. (2020). A learning theory of attachment: Unraveling the black box of attachment development. *Neurosci. Biobehav. Rev.* 113 287–298. 10.1016/j.neubiorev.2020.03.014 32276142

[B11] Bull R. Lee K. Koh I. H. C. Poon K. K. L. (2016). Confirmatory factor analysis of the strengths and difficulties questionnaire in Singaporean kindergartners. *Child* 42 109–116. 10.1111/cch.12288 26470606

[B12] Cheung H. S. (2021). Maternal sensitivity and warmth in Singapore: cultural insights from comparing the ainsworth and emotional availability scales. *Infant Child Dev.* 30:e2233.

[B13] Cooke J. E. Deneault A. A. Devereux C. Eirich R. Fearon R. M. P. Madigan S. (2022). Parental sensitivity and child behavioral problems: A meta-analytic review. *Child Dev.* 93 1231–1248. 35357693 10.1111/cdev.13764

[B14] De Wolff M. S. Van Ijzendoorn M. H. (1997). Sensitivity and attachment: a meta-analysis on parental antecedents of infant attachment. *Child Dev.* 68 571–591. 9306636

[B15] Dunn E. C. Busso D. S. Raffeld M. R. Smoller J. W. Nelson C. A. Doyle A. E. (2016). Does developmental timing of exposure to child maltreatment predict memory performance in adulthood? Results from a large, population-based sample. *Child Abuse Neglect.* 51 181–191. 10.1016/j.chiabu.2015.10.014 26585216 PMC4713298

[B16] Egger H. L. Pine D. S. Nelson E. Leibenluft E. Ernst M. Towbin K. E. (2011). The NIMH child emotional faces picture set (NIMH-ChEFS): A new set of children’s facial emotion stimuli. *Int. J. Methods Psychiatric Res*. 20, 145–156. 10.1002/mpr.343 22547297 PMC3342041

[B17] Ellis B. J. Bianchi J. Griskevicius V. Frankenhuis W. E. (2017). Beyond risk and protective factors: an adaptation-based approach to resilience. *Perspect. Psychol. Sci.* 12 561–587. 10.1177/1745691617693054 28679332

[B18] Fanselow M. S. Dong H.-W. (2010). Are the dorsal and ventral hippocampus functionally distinct structures? *Neuron* 65 7–19. 20152109 10.1016/j.neuron.2009.11.031 PMC2822727

[B19] Faul L. LaBar K. S. (2022). Mood-congruent memory revisited. *Psychol. Rev.* [Online ahead of print] 10.1037/rev0000394 36201828 PMC10076454

[B20] Fischl B. Salat D. H. Busa E. Albert M. Dieterich M. Haselgrove C. (2002). Whole brain segmentation: automated labeling of neuroanatomical structures in the human brain. *Neuron* 33 341–355. 11832223 10.1016/s0896-6273(02)00569-x

[B21] Fischl B. Salat D. H. Van der Kouwe A. J. Makris N. Ségonne F. Quinn B. T. (2004a). Sequence-independent segmentation of magnetic resonance images. *Neuroimage* 23(Suppl. 1), S69–S84. 10.1016/j.neuroimage.2004.07.016 15501102

[B22] Fischl B. Van der Kouwe A. Destrieux C. Halgren E. Ségonne F. Salat D. H. (2004b). Automatically parcellating the human cerebral cortex. *Cereb. Cortex* 14 11–22. 14654453 10.1093/cercor/bhg087

[B23] Frankenhuis W. E. Amir D. (2022). What is the expected human childhood? Insights from evolutionary anthropology. *Dev. a Psychopathol.* 34 473–497. 10.1017/S0954579421001401 34924077

[B24] Frankenhuis W. E. De Vries S. A. Bianchi J. Ellis B. J. (2020). Hidden talents in harsh conditions? A preregistered study of memory and reasoning about social dominance. *Dev. Sci.* 23:e12835. 10.1111/desc.12835 30985945 PMC7379268

[B25] Gee D. G. Gabard-Durnam L. J. Flannery J. Goff B. Humphreys K. L. Telzer E. H. (2013). Early developmental emergence of human amygdala-prefrontal connectivity after maternal deprivation. *PNAS Proc. Natl. Acad. Sci. U.S.A.* 110 15638–15643. 10.1073/pnas.1307893110 24019460 PMC3785723

[B26] Goodman A. Goodman R. (2009). Strengths and difficulties questionnaire as a dimensional measure of child mental health. *J. Am. Acad. Child Adolesc. Psychiatry* 48 400–403. 19242383 10.1097/CHI.0b013e3181985068

[B27] Goodman R. (1997). The strengths and difficulties questionnaire: a research note. *J. Child Psychol. Psychiatry* 38 581–586. 9255702 10.1111/j.1469-7610.1997.tb01545.x

[B28] Guillery-Girard B. Martins S. Deshayes S. Hertz-Pannier L. Chiron C. Jambaque I. (2013). Developmental trajectories of associative memory from childhood to adulthood: a behavioral and neuroimaging study. *Front. Behav. Neurosci.* 7:126. 10.3389/fnbeh.2013.00126 24098276 PMC3784827

[B29] Hannula D. E. Ranganath C. (2008). Medial temporal lobe activity predicts successful relational memory binding. *J. Neurosci.* 28 116–124. 18171929 10.1523/JNEUROSCI.3086-07.2008 PMC2748793

[B30] Hesse E. Main M. (2006). Frightened, threatening, and dissociative parental behavior in low-risk samples: Description, discussion, and interpretations. *Dev. Psychopathol.* 18 309–343. 10.1017/S0954579406060172 16600057

[B31] Hulley S. B. Cummings S. R. Browner W. S. Grady D. Newman T. B. (2013). *Designing clinical research: an epidemiologic approach*, 4th Edn. Philadelphia, PA: Lippincott Williams & Wilkins.

[B32] Iglesias J. E. Augustinack J. C. Nguyen K. Player C. M. Player A. Wright M. (2015). A computational atlas of the hippocampal formation using ex vivo, ultra-high resolution MRI: Application to adaptive segmentation of in vivo MRI. *Neuroimage* 115 117–137. 10.1016/j.neuroimage.2015.04.042 25936807 PMC4461537

[B33] Koo T. K. Li M. Y. (2016). A guideline of selecting and reporting intraclass correlation coefficients for reliability research. *J. Chiropr. Med.* 15 155–163. 27330520 10.1016/j.jcm.2016.02.012 PMC4913118

[B34] Kovacs M. Obrosky D. S. Sherrill J. (2003). Developmental changes in the phenomenology of depression in girls compared to boys from childhood onward. *J. Affect. Disord.* 74 33–48. 10.1016/s0165-0327(02)00429-9 12646297

[B35] Lambert H. K. Sheridan M. A. Sambrook K. A. Rosen M. L. Askren M. K. Mclaughlin K. A. (2017). Hippocampal contribution to context encoding across development is disrupted following early-life adversity. *J. Neurosci.* 37 1925–1934. 10.1523/JNEUROSCI.2618-16.2017 28093475 PMC5320618

[B36] Lawson M. Chae Y. Noriega I. Valentino K. (2021). Parent-child attachment security is associated with preschoolers’ memory accuracy for emotional life events through sensitive parental reminiscing. *J. Exp. Child Psychol.* 209:105168. 10.1016/j.jecp.2021.105168 33940484

[B37] Lee A. Poh J. S. Wen D. J. Tan H. M. Chong Y.-S. Tan K. H. (2019). Maternal care in infancy and the course of limbic development. *Dev. Cogn. Neurosci.* 2019:100714. 10.1016/j.dcn.2019.100714 31614256 PMC6974899

[B38] Lee J. K. Fandakova Y. Johnson E. G. Cohen N. J. Bunge S. A. Ghetti S. (2020). Changes in anterior and posterior hippocampus differentially predict item-space, item-time, and item-item memory improvement. *Dev. Cogn. Neurosci.* 41:100741. 10.1016/j.dcn.2019.100741 31826840 PMC6994624

[B39] Lee J. K. Wendelken C. Bunge S. A. Ghetti S. (2016). A time and place for everything: developmental differences in the building blocks of episodic memory. *Child Dev.* 87 194–210. 10.1111/cdev.12447 26493950 PMC4733390

[B40] LeMoult J. Gotlib I. H. (2019). Depression: A cognitive perspective. *Clin. Psychol. Rev.* 69 51–66. 29961601 10.1016/j.cpr.2018.06.008 PMC11884012

[B41] LoBue V. Thrasher C. (2015). The Child Affective Facial Expression (CAFE) set: validity and reliability from untrained adults. *Front. Psychol.* 5:1532. 10.3389/fpsyg.2014.01532 25610415 PMC4285011

[B42] Luby J. L. Barch D. M. Belden A. Gaffrey M. S. Tillman R. Babb C. (2012). Maternal support in early childhood predicts larger hippocampal volumes at school age. *Proc. Natl. Acad. Sci. U.S.A.* 109 2854–2859. 10.1073/pnas.1118003109 22308421 PMC3286943

[B43] Luby J. L. Belden A. Botteron K. Marrus N. Harms M. P. Babb C. (2013). The effects of poverty on childhood brain development: the mediating effect of caregiving and stressful life events. *JAMA Pediatr.* 167 1135–1142. 10.1001/jamapediatrics.2013.3139 24165922 PMC4001721

[B44] Luby J. L. Belden A. Harms M. P. Tillman R. Barch D. M. (2016). Preschool is a sensitive period for the influence of maternal support on the trajectory of hippocampal development. *Proc. Natl. Acad. Sci. U.S.A.* 113 5742–5747. 10.1073/pnas.1601443113 27114522 PMC4878487

[B45] Madigan S. Bakermans-Kranenburg M. J. Van Ijzendoorn M. H. Moran G. Pederson D. R. Benoit D. (2006). Unresolved states of mind, anomalous parental behavior, and disorganized attachment: a review and meta-analysis of a transmission gap. *Attach Hum. Dev.* 8 89–111. 10.1080/14616730600774458 16818417

[B46] Main M. (1981). “Avoidance in the service of attachment,” in *Behavioral development*, eds Immelman K. B. G. Petrinovitch L. Main M. (Cambridge: Cambridge University Press).

[B47] Mickley S. K. R. Anderson A. J. Brasher K. I. Brehmer T. S. (2017). Cortisol and stimulus-induced arousal level differentially impact memory for items and backgrounds. *Cogn. Emot.* 31 325–338. 10.1080/02699931.2015.1111197 26577049

[B48] Moran G. Forbes L. Evans E. Tarabulsy G. M. Madigan S. (2008). Both maternal sensitivity and atypical maternal behavior independently predict attachment security and disorganization in adolescent mother-infant relationships. *Infant Behav. Dev.* 31 321–325. 10.1016/j.infbeh.2007.12.012 18282606

[B49] Mu S. H. Yuan B. K. Tan L. H. (2020). Effect of gender on development of hippocampal subregions from childhood to adulthood. *Front. Hum. Neurosci.* 14:611057. 10.3389/fnhum.2020.611057 33343321 PMC7744655

[B50] Olsen R. K. Moses S. N. Riggs L. Ryan J. D. (2012). The hippocampus supports multiple cognitive processes through relational binding and comparison. *Front. Hum. Neurosci.* 6:146. 10.3389/fnhum.2012.00146 22661938 PMC3363343

[B51] Pederson D. R. Moran G. Bento S. (2013). *The Maternal Behavior Q-sort: Assessing Maternal Sensitivity and the Quality of Mother-Infant Interaction.* London: University of Western Ontario.

[B52] Peltola M. J. Forssman L. Puura K. Van Ijzendoorn M. H. Leppänen J. M. (2015). Attention to faces expressing negative emotion at 7 months predicts attachment security at 14 months. *Child Dev.* 86 1321–1332. 10.1111/cdev.12380 26011101 PMC5008154

[B53] Perry R. E. Finegood E. D. Braren S. H. Dejoseph M. L. Putrino D. F. Wilson D. A. (2018). Developing a neurobehavioral animal model of poverty: Drawing cross-species connections between environments of scarcity-adversity, parenting quality, and infant outcome. *Dev. Psychopathol.* 31 399−418. 10.1017/S095457941800007X 29606185 PMC6168440

[B54] Price R. B. Duman R. (2020). Neuroplasticity in cognitive and psychological mechanisms of depression: an integrative model. *Mol. Psychiatry* 25 530–543. 31801966 10.1038/s41380-019-0615-x PMC7047599

[B55] Rao H. Betancourt L. Giannetta J. M. Brodsky N. L. Korczykowski M. Avants B. B. (2010). Early parental care is important for hippocampal maturation: evidence from brain morphology in humans. *Neuroimage* 49 1144–1150. 10.1016/j.neuroimage.2009.07.003 19595774 PMC2764790

[B56] Richmond J. L. Pan R. (2013). Thinking about the future early in life: the role of relational memory. *J. Exp. Child Psychol.* 114 510–521. 23267734 10.1016/j.jecp.2012.11.002

[B57] Rifkin-Graboi A. Goh S. K.-Y. Chong H. J. Tsotsi S. Sim L. W. Tan K. H. (2021). Caregiving adversity during infancy and preschool cognitive function: adaptations to context? *J. Dev. Origins Health Dis.* 12 890–901. 10.1017/S2040174420001348 33436135

[B58] Rifkin-Graboi A. Khng K. H. Cheung P. Tsotsi S. Sun H. Kwok F. (2019). Will the future BE POSITIVE? Early life experience as a signal to the developing brain pre school entry. *Learning* 5 99–125.

[B59] Rifkin-Graboi A. Kong L. Sim L. W. Sanmugam S. Broekman B. F. Chen H. (2015). Maternal sensitivity, infant limbic structure volume and functional connectivity: a preliminary study. *Transl. Psychiatry* 5:e668. 10.1038/tp.2015.133 26506054 PMC4930120

[B60] Rifkin-Graboi A. Ngoh G. (2022). “Parenting, challenges, brain development and attachment strategies,” in *The cambridge handbook of parenting (cambridge handbooks in psychology)*, eds Morris A. Mendez J. Smith (Cambridge: Cambridge University Press), 29–49.

[B61] Rifkin-Graboi A. Quan J. Richmond J. Goh S. K. Y. Sim L. W. Chong Y. S. (2018). Greater caregiving risk, better infant memory performance? *Hippocampus* 28 497–511. 10.1002/hipo.22949 29663599

[B62] Schilling T. M. Kolsch M. Larra M. F. Zech C. M. Blumenthal T. D. Frings C. (2013). For whom the bell (curve) tolls: cortisol rapidly affects memory retrieval by an inverted U-shaped dose-response relationship. *Psychoneuroendocrinology* 38 1565–1572. 10.1016/j.psyneuen.2013.01.001 23374327

[B63] Ségonne F. Dale A. M. Busa E. Glessner M. Salat D. Hahn H. K. (2004). A hybrid approach to the skull stripping problem in MRI. *Neuroimage* 22 1060–1075. 10.1016/j.neuroimage.2004.03.032 15219578

[B64] Shackman J. E. Shackman A. J. Pollak S. D. (2007). Physical abuse amplifies attention to threat and increases anxiety in children. *Emotion* 7 838–852. 10.1037/1528-3542.7.4.838 18039053

[B65] Sled J. G. Zijdenbos A. P. Evans A. C. (1998). A nonparametric method for automatic correction of intensity nonuniformity in MRI data. *IEEE Trans. Med. Imaging* 17 87–97. 9617910 10.1109/42.668698

[B66] Strange B. A. Witter M. P. Lein E. S. Moser E. I. (2014). Functional organization of the hippocampal longitudinal axis. *Nat. Rev. Neurosci.* 15 655–669. 25234264 10.1038/nrn3785

[B67] Tsotsi S. Borelli J. Binte Abdulla N. Tan H. M. Sim L. W. Sanmugam S. (2018). Maternal sensitivity during infancy and the regulation of startle in preschoolers. *Attachment Hum. Dev.* 22 207−224. 10.1080/14616734.2018.1542737 30406719

[B68] Vandevivere E. Braet C. Bosmans G. Mueller S. C. De Raedt R. (2014). Attachment and children’s biased attentional processing: Evidence for the exclusion of attachment-related information. *PLoS One* 9:e0103476. 10.1371/journal.pone.0103476 25061662 PMC4111605

[B69] Wang Q. Zhang H. Wee C. Y. Lee A. Poh J. S. Chong Y. S. (2019). Maternal sensitivity predicts anterior hippocampal functional networks in early childhood. *Brain Struct. Funct.* 224 1885–1895. 10.1007/s00429-019-01882-0 31055646

[B70] Young E. S. Frankenhuis W. E. Delpriore D. J. Ellis B. J. (2022). Hidden talents in context: Cognitive performance with abstract versus ecological stimuli among adversity-exposed youth. *Child Dev.* 93 1493–1510. 10.1111/cdev.13766 35404500 PMC9543758

